# Clinical and microbiological characterization of *Salmonella spp*. isolates from patients treated in a university hospital in South America between 2012–2021: a cohort study

**DOI:** 10.1186/s12879-023-08589-y

**Published:** 2023-09-25

**Authors:** Fernando Rosso, David E. Rebellón-Sánchez, Julio Llanos-Torres, Leidy Johanna Hurtado-Bermudez, Laura Ayerbe, John Harold Suárez, Nicolás Orozco-Echeverri, Cristhian Camilo Rojas-Perdomo, Isabel Lucia Zapata-Vasquez, Jaime Patiño-Niño, Luis Gabriel Parra-Lara

**Affiliations:** 1 Fundación Valle del Lili, Centro de Investigaciones Clínicas (CIC), Carrera 98 #18-49, 760031 Cali, Colombia; 2 Fundación Valle del Lili, Cali, Departamento de Medicina Interna, Servicio de Enfermedades Infecciosas, Carrera 98 #18-49, 760031 Cali, Colombia; 3 Universidad Icesi, Facultad de Ciencias de la Salud, Calle 18 No. 122-135, Cali, 760031 Colombia; 4 Fundación Valle del Lili, Cali, Departamento de Pediatría, Servicio de Enfermedades Infecciosas, Carrera 98 #18-49, 760031 Cali, Colombia

**Keywords:** Salmonella, Salmonella typhi, Salmonella infections, Foodborne diseases

## Abstract

**Background:**

Salmonellosis is a major cause of morbidity and mortality and one of the most frequent etiologies of diarrhea in the world. Mortality due to Salmonellosis in Latin America still poorly understood, and there is a lack of studies that evaluate resistance and clinical manifestations. The aims of this study were to characterize patients infected with *Salmonella spp*. seen in a university hospital in Colombia between 2012 and 2021, to evaluate trends in antibiotic resistance and to determine the proportion of overall mortality and related factors.

**Methods:**

Retrospective observational study. All patients with microbiological diagnosis of *Salmonella spp*. were included. The sociodemographic, clinical and microbiological characteristics were described, and the proportion of antibiotic resistant isolates per year was estimated. The prevalence of mortality according to age groups was calculated. Log binomial regression models were used to establish factors associated with mortality.

**Results:**

Five hundred twenty-two patients were analyzed. Salmonellosis accounted for 0.01% of all medical consultations. The median age was 16 years old. The most common clinical presentation was gastroenteric syndrome (77.1%) and symptoms included diarrhea (79.1%), fever (66.7%), abdominal pain (39.6%) and vomiting (35.2%). Of the *Salmonella spp.* isolates, 78.2% were not classified, 19.1% corresponded to non-typhoidal Salmonella and 2.7% to *Salmonella typhi*. Mortality occurs in 4.02% of the patients and was higher in patients with hematologic malignancy (11.6%). When analyzing by age group, the proportion of deaths was 2.8% in patients aged 15 years or younger, while in those older than 15 years it was 5.4%. Factors associated to mortality where bacteremia (aPR = 3.41 CI95%: 1.08—10.76) and to require treatment in the ICU (aPR = 8.13 CI95%: 1.82—37.76). In the last 10 years there has been a steady increase in resistance rates to ciprofloxacin, ampicillin, ampicillin/sulbactam and ceftriaxone, reaching rates above 60% in recent years.

**Conclusions:**

Despite improved availability of antibiotics for the treatment of salmonellosis in the past decades, mortality due to salmonellosis continues occurring in children and adults, mainly in patients with hematological malignancies and bacteremia. Antibiotic resistance rates have increased significantly over the last 10 years. Public health strategies for the control of this disease should be strengthened, especially in vulnerable populations.

**Supplementary Information:**

The online version contains supplementary material available at 10.1186/s12879-023-08589-y.

## Background

Salmonellosis is an important cause of morbidity and mortality and one of the most frequent etiologies of infectious diarrheal disease in the world [1]. Chicken meat, eggs and dairy products are the foods most associated with transmission of the disease [1, 2]. The most common symptoms include abdominal pain, diarrhea, vomiting, fever and in some cases, severe systemic manifestations that can lead to death [3]. Salmonella is a rod-shaped gram-negative facultative pathogen capable of infecting many hosts and various cell types. Its ability to survive and thrive in all the cell population indicates how the pathogen gets an upper hand in the infected host [4].

*Salmonella enterica* is a species consisting of more than 2,600 different serovars that can be divided into typhoidal (*Salmonella typhi*) and non-typhoidal Salmonellae (NTS) serovars. *Salmonella typhi* serovar causes typhoid fever. Each year, between 128,000 and 161,000 people die worldwide due to this disease [5]. Symptoms consist of prolonged fever, fatigue, headache, nausea, abdominal pain, constipation, and diarrhea. In severe cases, complications may include confusion, neurological deterioration, gastrointestinal perforations, myocarditis, hepatitis, sepsis, shock, disseminated intravascular coagulation, and death [6]. On the other hand, enteric fever caused by typhoidal serovars differs dramatically from the gastroenteritis normally associated with NTS. Patients infected by typhoidal serovars most typically present with a gradual onset of sustained fever, chills, abdominal pain, hepatosplenomegaly, rash, nausea, anorexia, diarrhea or constipation, headache, and a dry cough. Individuals infected with NTS have self-limiting, acute gastroenteritis, and diarrhea [7].

In Colombia, there have been documented efforts to estimate the prevalence and characteristics of diseases caused by *Salmonella spp*. From 1997 to 2018, the National Institutes of Health (Instituto Nacional de Salud—INS) collected information on the antimicrobial susceptibility profile of *Salmonella spp.*, and recorded an increase in resistance to beta-lactams and quinolones from 2000 to 2012 [8, 9]. This is important, considering that 859 outbreaks of foodborne diseases were reported by the INS in 2021 and about 10% were caused by *Salmonella spp. *[10].

Although this microorganism has been identified as a cause of various infections, such as bacteriemia, urinary tract infections, and osteoarticular infections, there is limited information on its species distribution and resistance patterns in Latin America. Furthermore, there is a lack of data on clinical outcomes, such as mortality rates. As a result, further studies are necessary in Latin America to determine the prevalence of Salmonellosis in hospitals, the frequency of resistant strains, the associated risk factors, and the clinical outcomes [11]. The aims of this study were to characterize patients infected with *Salmonella spp*. seen in a university hospital in South America between 2012 and 2021, to evaluate trends in antibiotic resistance, and to determine the proportion of overall mortality and related factors.

## Methods

### Study design, inclusion criteria, and data collection.

Retrospective cross-sectional study describing the clinical and microbiological characteristics of patients infected by *Salmonella typhi* and NTS serovars attended from January 2012 to December 2021 at the Fundación Valle del Lili (FVL), Cali, Colombia. All patients with positive culture isolation for *Salmonella spp.* were included regardless of admission diagnosis, age, sex or comorbidities. The decision to take cultures was based on the personal clinical judgment of each institution's physician in outpatient, inpatient, and intensive care services. Not all patients seen in the clinic underwent culture to identify *Salmonella spp*.

### Setting and ethical considerations

The study was conducted at FVL. It is a university hospital that serves as a reference center for the Pacific region and part of the Andean and Amazon region in Colombia.

The study was conducted in accordance with the Declaration of Helsinki and the Council for International Organizations of Medical Sciences recommendations. It was approved by the FVL research ethics board committee (Approval Act 17–2002). As research without risk according to Colombian law, it was considered exempt from applying informed consent (Resolution 8430 of 1993).

### Variables and data management

Sociodemographic, clinical, microbiological and treatment variables were collected including age group, sex, comorbidities, clinical presentation, isolated salmonella and clinical outcomes such as overall mortality, hospitalization requirement, in-hospital complications and ICU requirement. All the clinical data and laboratory tests were consulted from the patient medical records and was stored in an electronic case report form using in-home software developed at FVL (“BD Clinic”) for this purpose.

Persistent diarrhea was considered when the symptom lasted 14 days or more [12], and chronic diarrhea when it lasted 4 weeks or more [13]. A recent trip was defined as any trip out of town for more than 3 days for any reason, in the last 3 months to the development of symptoms. The use of antibiotics in the last month, took into account that there was a record in the clinical history of each patient. Exposure to immunosuppressive therapy was defined as having been exposed to corticosteroids at any dose, colchicine, antimalarials such as chloroquine or hydroxychloroquine, inhibitors of prostaglandin formation such as sulfasalazine, monoclonal antibodies that impact the functioning of the immune system, as well as other medications such as dapsone, methotrexate, mycophenolate, azathioprine, or any other drug that interferes with innate or adaptive immunity at the time of symptom development or one month prior to it.

ESBL were defined as the presence of a strain that produced β-lactamases capable of hydrolyzing certain types of antibiotics such as penicillins, third-generation cephalosporins, and monobactams. The ESBL-producing bacterial isolates were phenotypically characterized using the New VITEK 2 Test for Rapid Detection of ESBL Production in Enterobacteriaceae Isolates [14]. Mortality was considered only in those who required hospitalization in our institution and died during the same hospitalization in which *Salmonella spp.* isolation was performed. To evaluate resistance trends, resistant isolates were defined as those reported in the antibiogram as having intermediate or resistant sensitivity.

### Laboratory procedures from which the data was obtained

All microbiological data were extracted directly from institutional reports. The institutional protocols used for isolation and identification of *Salmonella spp.* serovars are summarized below. Laboratory procedures were divided into two phases: isolation and subspecies identification. The consistency of the fecal samples was evaluated to determine the culture process. Liquid samples were seeded on culture media for *Campylobacter spp., Vibrio cholerae*, blood agar, MacConkey agar (MAC), hektoen enteric agar (HEA), and selenite agar and incubated at 37 °C. Non-liquid samples were seeded on blood agar, MAC agar, HEA agar and selenite agar. Salmonellosis was considered possible when growth was identified on blood agar, MAC agar and selenite agar. To confirm the diagnosis, a colony of selenite agar was used for subspecies identification.

Secretion samples (e.g., orotracheal secretion) were seeded on blood agar, MAC agar and Columbia Nalidixic Acid (CNA) agar. Colonies suggestive of *Salmonella spp.* were obtained from blood agar and MAC agar media for subspecies identification. Sterile fluid samples (e.g., cerebrospinal fluid, urine) were seeded on chocolate agar and PHI-enriched culture medium. Finally, the blood culture sample was incubated at 37 °C in automated BioMerieux equipment. Positive samples (growth alert) were evaluated by Gram staining and culture on blood agar and MAC agar.

Once the colony was obtained, the VITEK®MS automated equipment was used to identify the bacterial genus and species by mass spectrometry, which has reported a sensitivity of over 98% for genus and species identification in enterobacterial isolates [15]. Sometimes *Salmonella spp.* was identified without determining the subspecies, so it was proceeded to confirmation using VITEK®2, which has a sensitivity of 98.1% with a positive predictive value of 99.3% and a specificity of 99.7% with a negative predictive value of 99.3% [14], if the subspecies still not identified, it was used a third platform (MicroScan WalkAway plus system). When identification was not achieved with any of the three equipments, it was registered as *Salmonella spp.*

After confirmation of the species, an antibiogram was performed and resistance was determined according to the current cut-off points recommended by The Clinical & Laboratory Standards Institute (CLSI) [16]. In addition, the CARBA NP test (CNPt) or the rapidec test (bioMerieux), and the NG-test CARBA 5 were performed. CNPt, is a novel phenotypic method developed for carbapenemase detection based on in vitro hydrolysis of imipenem by a bacterial lysate, which is detected by changes in pH values [17]. While NG-test CARBA 5 a rapid in vitro multiplex immunoassay for the phenotypic detection and differentiation of five common carbapenemase families [18].

### Sample size

Convenience sampling was performed including all participants with positive *Salmonella spp.* culture regardless of age or clinical status. Outpatients, inpatients, and intensive care patients were all included in the study. All participants with Salmonellosis were identified from the total number of patients treated at the Fundacion Valle de Lili, regardless of their reason for consultation.

### Statistical analysis

For descriptive analysis, qualitative variables were presented as frequencies and percentages. QQ-plot and Shapiro–Wilk were used to evaluate the assumption of normal distribution of quantitative variables. All quantitative variables had a non-normal distribution and were summarized as median and interquartile range (IQR). Comparison between groups was performed using the χ2 test or Fisher's exact test for categorical variables, and the Mann–Whitney U test. A significance level of 5% was applied to show the significance of the variables.

In addition, univariate and multivariate Log-Binomial regressions were constructed to identify the factors associated with mortality according to age group. For this purpose, the reference value of age reported in previous studies was considered [11]. All variables with a p value less than 0.15 in the bivariate analysis were entered into the regression models. The selection of variables for the final model was made by statistical and biological significance of the variables. All analyses were performed with STATA® (version 17.0, StataCorp LP, College Station, TX).

## Results

**Fig. 1:**
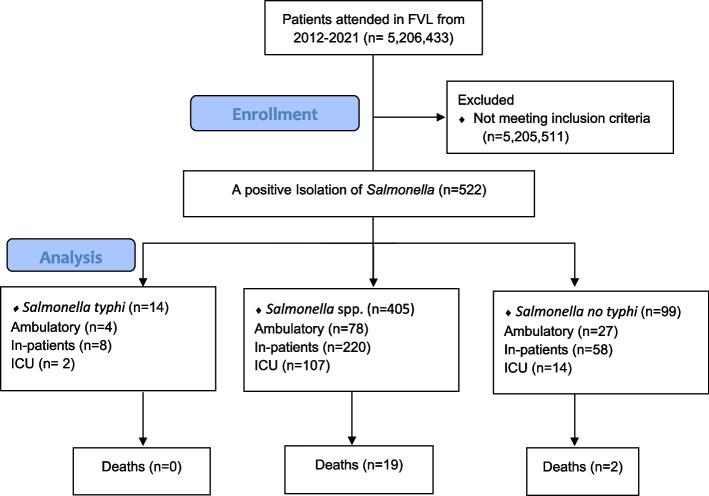
Flowchart of the study

A total of 5,206,433 patients were attended at the hospital between 2012 and 2021 Among all patients seen, the proportion of salmonellosis cases was 0.01% (*n* = 522) over a 10-year period. In total, nearly 1,592,960 cultures were performed on patients during the study period. Of these, the percentage of cultures positive for Salmonellosis was 0.03% among all cultures ordered at the clinic. 405 isolations were reported as *Salmonella spp* in culture, 99 as NTS and 14 as *Salmonella typhi*. A total of 21 deaths occurred. The enrollment flowchart, management setting and mortality by group are summarized in Fig. 1.

Approximately 50% of patients were under 16 years of age (Me 16.5 (IQR: 1–50) and the disease was more frequent in females. The most frequent symptoms included diarrhea (79.1%), fever (66.7%), abdominal pain (39.6%) and vomiting (35.2%). It is noteworthy that a higher proportion of patients with abdominal pain was recorded in those aged 15 years and older than in those aged 15 years and younger (52.3% vs. 26.5%, *p* < 0.001), while fever and diarrhea were more commonly recorded in children below 15 years of age (*p* = 0.005 and *p* < 0.001, respectively). Moreover, bacteremia was more common in the older group (19.3% vs. 26.8%, *p* = 0.04) Furthermore, a higher frequency of comorbidities was recorded in participants older than 15 years (*p* < 0.05 in the prevalence of cancer, diabetes, liver disease, chronic renal disease and others).

Among the factors reported by patients, 20.3% were receiving immunosuppressive therapy, 11.3% had received antibiotics in the last month, 9.2% started antibiotics after food intake and 3.7% reported recent travel. A total of 372 patients (71.25%) received some type of empirical antibiotic treatment. The most frequently used empirical antibiotic treatment during hospital stay was ceftriaxone (35.1%), followed by piperacillin/Tazobactam (10.2%) and ciprofloxacin (7.8%). Interestingly, a higher proportion of resistant isolates was observed in children under 15 years of age than in the population aged 15 years and older (28.76% vs 21.10%, *p* = 0.055). Of the total of patients, 79.1% were hospitalized, 24% required intensive care (ICU), 11.7% developed complications derived from salmonellosis and the overall mortality was 4.1%. In-hospital complications and ICU requirement were more frequent in those older than 15 years of age. Table 1 summarizes the sociodemographic, clinical and microbiological characteristics and outcomes in the study population by age group.

**Table 1:** Sociodemographic, clinical characteristics and outcomes of patients (*n* = 522)

Characteristics	Total, *n* = 522	Age group		*p*-value
		** ≤ 15 years old,** ***n*** **= 257**	** > 15 years old,** ***n*** **= 265**	
Female sex, n (%)	286	135 (52.5)	151 (57.0)	0.307↟
**Signs and symptoms, n (%)**				
Fever	345	184 (72.7)	161 (61.0)	0.005↟
Diarrhea	379	205 (80.7)	174 (65.9)	< 0.001↟
Self-limited	336	214 (81.1)	230 (90.3)	0.259↟
Persistent	42	25 (11.0)	17 (6.4)	0.247↟
Chronic	26	18 (7.9)	8 (3.3)	0.069↟
Emesis	182	89 (35.2)	93 (35.2)	0.991↟
Abdominal pain	205	67 (26.5)	138 (52.3)	< 0.001↟
Dehydration	156	76 (30.0)	80 (30.3)	0.948↟
Anorexy	60	29 (11.5)	31 (11.7)	0.921↟
Loss of appetite	131	61 (24.1)	70 (26.5)	0.530↟
Lethargy	52	21 (8.3)	31 (11.7)	0.193↟
Respiratory symptoms	79	46 (18.2)	33 (12.5)	0.073↟
**Medical history, n (%)**				
Gastric surgery	31	9 (3.5)	22 (8.3)	0.002↟
HIV	7	1 (0.4)	6 (2.3)	0.123^
Cancer history				
Solid organ cancer	36	12 (4.7)	24 (9.1)	0.052↟
Hematological cancer	60	16 (6.3)	44 (16.6)	< 0.001↟
Chemotherapy last three months	51	15 (5.9)	36 (13.6)	0.003↟
Solid organ transplant recipient	35	10 (3.9)	25 (9.4)	0.013↟
Bone marrow transplant recipient	21	4 (1.6)	17 (6.4)	0.006^
Inflammatory bowel disease	9	5 (2.0)	4 (1.5)	0.747^
Chronic kidney disease	50	13 (5.1)	37 (14.0)	0.001↟
Immunosuppressive therapy	105	34 (13.4)	71 (26.8)	< 0.001↟
Pernicious anemia	10	0	10 (3.8)	0.014↟
Sickle cell disease	3	1 (0.4)	2 (0.7)	1.000^
Liver disease	27	7 (2.8)	20 (7.6)	0.014↟
Type 2 diabetes	32	6 (2.4)	26 (9.8)	< 0.001^
**Exposure, n (%)**				
Foodborne illness	48	17 (6.7)	31 (11.7)	0.049↟
Travel in the last 3 months	19	14 (5.5)	5 (1.9)	0.028↟
Antibiotic use in the last 30 days	73	38 (15.0)	35 (13.2)	0.566↟
**Clinical presentation, n (%)**				
Gastroenteric syndrome	400	214 (84.3)	186 (70.2)	< 0.001↟
Bacteremia	120	49 (19.3)	71 (26.8)	0.043↟
Urinary tract infection	30	6 (2.4)	24 (9.1)	0.001↟
Other^a^	31	13 (5.1)	18 (6.8)	0.402↟
**Microbiological characteristics, n (%)**				
Type of sample				
Blood culture	109	42 (16.5)	67 (25.4)	< 0.001↟
Stool culture	361	198 (78.0)	163 (61.7)	
Urine culture	27	6 (2.4)	21 (8.0)	
Other	21	8 (3.1)	13 (4.9)	
*Salmonella spp.* and *salmonella* serovars				
*Salmonella spp*	405	208 (81.8)	197 (74.6)	0.189^
*Salmonella choleraesuis*	94	39 (15.4)	55 (20.8)	
*Salmonella enteritidis*	5	1 (0.4)	4 (1.5)	
*Salmonella typhi*	14	6 (2.4)	8 (3.1)	
Antibiogram performed	470	233 (90.66)	237 (89.43)	0.640↟
Resistance to any antibiotic	117	67 (28.76)	50 (21.10)	0.055↟
ESBL	35	15 (8.8)	20 (9.9)	0.052↟
Positive CNPt	2	2 (1.4)	0 (0)	0.068^
Positive NG-CARBA-5	1	1 (0.7)	0 (0)	0.074^
**Clinical outcomes, n (%)**				
Overall mortality, *n* = 513	21	7 (2.8)	14 (5.4)	0.139↟
Hospitalization	412	198 (77.3)	214 (80.7)	0.339↟
In-hospital complications	60	17 (7.1)	43 (16.7)	< 0.001↟
ICU	125	41 (16.0)	84 (31.7)	< 0.001↟

*ESBL*Extended spectrum β-lactamases, *HIV*Human immunodeficiency virus, *ICU*Intensive care unit, *CNPt*CARBA-NP test

^a^ Other clinical presentations include meningitis, arthritis, osteomyelitis, hepatobiliary involvement, bone marrow infection, peritonitis and soft tissue infections

↟ Chi-square test

^ Fisher's exact test

Of the *Salmonella spp.* isolates, 78.2% were not classified, 19.1% corresponded to NTS and 2.7% to *Salmonella typhi*. A total of 69.7% were isolated from stool culture, 21.0% from blood culture, 5.2% from urine and 4.1% from other less frequent sites such as bones, soft tissue, bone marrow, peritoneal fluid.

A high proportion of resistance to ampicillin (21.19%), trimethoprim / sulfamethoxazole (TMP-SMX) (18.29%), nalidixic acid (16.77%) and ciprofloxacin (14.13%) was recorded in the *Salmonella spp.* isolates, with extended-spectrum beta-lactamase (ESBL) phenotype in 13.13% of isolates. When analyzed by subspecies, it was recorded that among NTS isolates, 59.09% were resistant to ciprofloxacin, 30.53% to ampicillin, 16.67% to ampicillin/sulbactam, 14.29% to Ceftriaxone, and 11.7% to TMP-SMX. For Salmonella typhi, the highest resistances were to ceftriaxone (60%) and TMP/SMX (22.22%). ESBL-producing bacteria was higher in *Salmonella typhi* isolates (42.86%) than in NTS isolates (9.26%). Table S1 summarizes the proportion of resistant isolates by species and antibiotic during the study period.

**Fig. 2:**
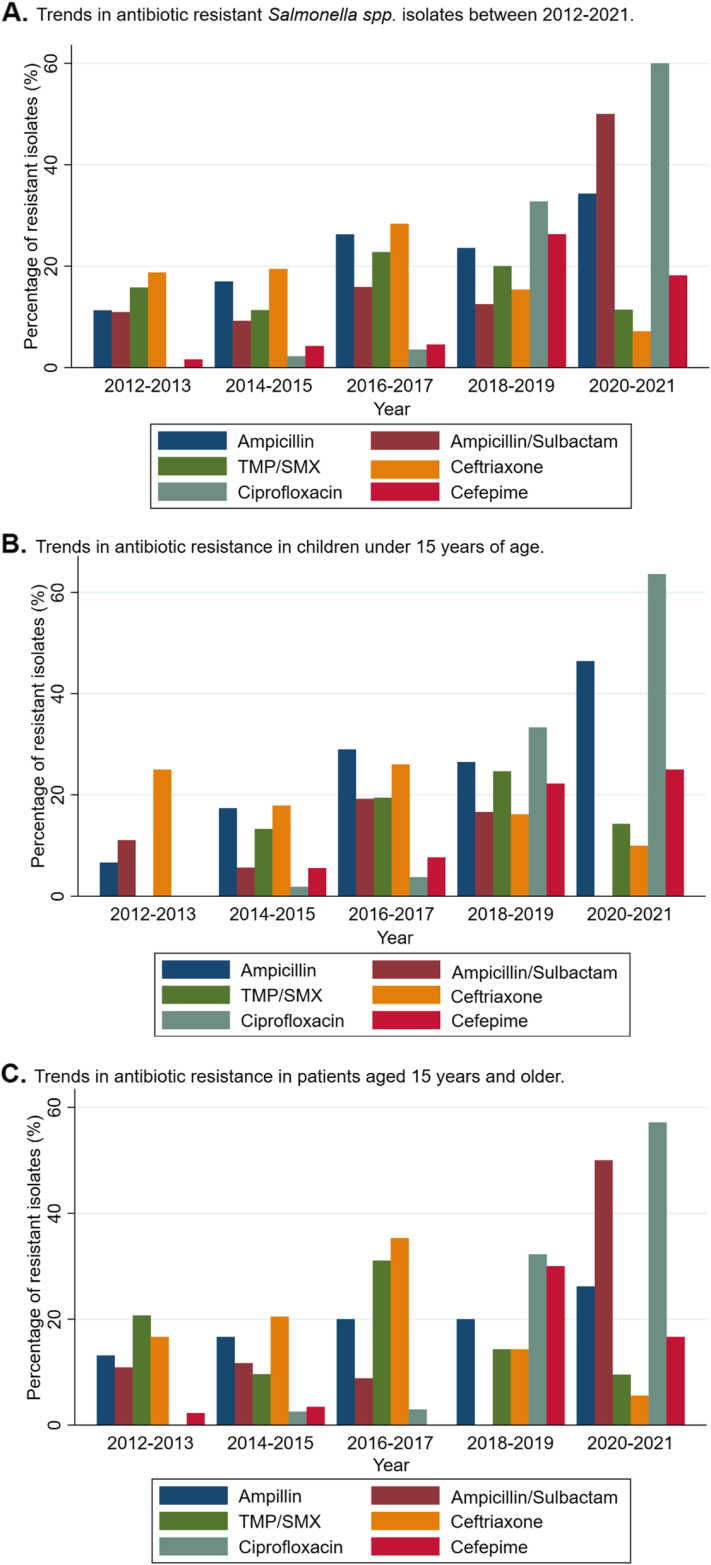
Trends in antibiotic resistant *Salmonella spp.* isolates between 2012–2021. **A** Overall study population (*n* = 470). **B** Below 15 years of age (*n* = 233). **C** Above 15 years of age (*n* = 237). Number of isolates with resistance testing for each antibiotic: Ampicillin, *n* = 457; Ampicillin/sulbactam, *n* = 313; TMP/SMX: trimethoprim / sulfamethoxazole, *n* = 431; Ceftriaxone, *n* = 261; Ciprofloxacin, *n* = 431; Cefepime, *n* = 219

Between 2012 and 2021, an alarming increase was observed in the percentages of isolates resistant per year to ciprofloxacin, ampicillin/sulbactam, ampicillin and cefepime. Figure 2 shows the trends in resistance to each of the antibiotics used in the treatment of salmonellosis by group of analysis.

Ciprofloxacin went from having no resistant isolates in the 2012–2013 period to having up to 60% of resistant species between 2020 and 2021, with the increase being more marked in patients under 15 years of age. Similarly, ampicillin/sulbactam resistance increased from less than 15% to almost 50% in recent years, especially in patients aged 15 years and older. Ampicillin went from having just under 15% in 2012–2013 to over 35% of resistant isolates between 2020–2021, while cefepime to which resistance was reported in less than 3% of cases in 2012–2013 rose to nearly 30% in the 2018–2019 period and an upward trend since the beginning of the follow-up period. For antibiotics such as TMP-SMX and Ceftriaxone, a more constant resistance pattern was recorded for both age groups with the highest pike between 2016—2017.

Strikingly, ESBL infections showed a significant upward trend between 2012 and 2020, especially in isolates from patients aged 15 years or younger, with an apparent decline in 2021, which correlates with a lower number of isolates made during the COVID-19 pandemic period.

The proportion of deaths was similar in both sexes. Overall mortality in patients aged 15 years or younger was 2.8%, while in those older than 15 years it was 5.4% (*p* = 0.13) Having hematological cancer (*p* < 0.1), gastroenteric syndrome, bacteriemia, ESBL infection and presenting in-hospital complications requiring ICU appeared to be related to a higher risk of mortality (*p* < 0.05). Other antecedents such as diabetes, liver disease, having traveled, having been involved in a foodborne disease outbreak, presenting symptoms such as fever and/or vomiting, and having a certain serovar of salmonella do not appear to be associated with a higher risk of presenting mortality (*p* > 0.05). Table 2 compares the sociodemographic, clinical and microbiological characteristics of the patients according to mortality group and are discriminated by age.

**Table 2:** Comparative analysis of overall mortality by clinical groups and variables of interest

Characteristics	Below 15 years of age		*p*-value	Above 15 years of age		*p*-value
	**Alive, *n* = 245**	**Dead, *n* = 7**		**Alive. *n* = 247**	**Dead, *n* = 14**	
**Gender**						
*Female*	127 (96.5)	4 (3.5)	1.000^	142 (96.0)	6 (4.0)	0.406^
**Medical history**						
Inflammatory bowel disease	3 (75.0)	1 (25.0)	0.337	3 (75.0)	1 (25.0)	0.199^
Hematological cancer	15 (100.0)	0	1.000^	38 (88.4)	5 (11.6)	0.061^a^
Chronic kidney disease	12 (92.3)	1 (7.7)	0.311^	32 (88.9)	4 (11.1)	0.110^
Liver disease	7 (100.0)	0	1.000^	18 (90.0)	2 (10.0)	0.292^
Chemotherapy last three months	15 (100.0)	0	1.000^	33 (91.7)	3 (8.3)	0.419^
Type 2 diabetes	6 (100.0)	0	1.000^	22 (91.7)	2 (8.3)	0.375^
Immunosuppressive therapy	33 (97.1)	1 (2.9)	1.000^	66 (94.3)	4 (5.7)	1.000^
**Exposure**						
Antibiotic use in the last 30 days	38 (100)	0	0.599^	30 (88.2)	4 (11.8)	0.093^
Foodborne illness	17 (100.0)	0	0.470^	29 (93.5)	2 (6.5)	0.676^
Travel in the last 3 months	14 (100)	0	1.000^	5 (100)	0	1.000^
**Clinical presentation**						
Bacteremia	47 (97.9)	1 (2.1)	1.000^	59 (85.8)	10 (14.5)	< 0.001^
Gastroenteric syndrome	208 (97.6)	5 (2.4)	0.296^	179 (97.3)	5 (2.7)	0.004^a^
**Characteristics of diarrhea**						
Persistent	19 (90.5)	2 (9.5)	0.135^	20 (95.2)	1 (4.8)	1.000^
Chronic	12 (100)	0	1.000^	14 (100)	0	1.000^
**Microbiology**						
Resistance to any antibiotic	67 (100)	0	0.581^	46 (92)	4 (8.00)	0.481^
ESBL	31 (93.9)	2 (6.1)	0.201^	16 (94.1)	1 (5.9)	1.000^
Positive CNPt	1 (100)	0	1.000^	1 (100)	0	1.000^
Positive CARBA-5	0	0		1 (100)	0	1.000^
***Salmonella spp*. and salmonella serovars**						
*Salmonella spp.*	199 (96.6)	7 (3.4)		182 (93.8)	12 (6.2)	
NTS	40 (100)	0		57 (96.4)	2 (3.6)	0.608^
*Salmonella Typhi*	6 (100)	0		8 (100)	0	
**Outcomes**						
ICU	36 (92.3)	3 (7.7)	0.077^	69 (85.2)	12 (14.8)	< 0.001^
Hospitalization	189 (97.4)	5 (2.6)	0.663^	197 (93.8)	13 (6.2)	0.316^

*NTS* Non-typhoideal salmonellae, *CNPt* CARBA-NP test

^a^ Chi-square test

^ Fisher's f-test

When performing univariate and multivariate analysis, no variables associated with mortality were found in patients under 15 years of age. While in the group older than 15 years of age, an association was found with bacteremia (aPR 3.41 CI95%: 1.08—10.76) and ICU requirement (aPR 8.13 CI95%: 1.82—37.76) (Table 3).

**Table 3:** Factors associated with mortality in patients older than 15 years of age

Characteristics	Patients above 15 years of age	
	**PR**	**aPR**
Hematological cancer	2.79 CI95%: 0.98—7.92	1.58 CI95%: 0.59—4.22
Chronic kidney disease	2.46 CI95%: 0.81—7.45	-
Antibiotic use in the last 30 days	2.62 CI95%: 0.87—7.92	-
Gastroenteric syndrome	0.23 CI95%: 0.08—0.68	-
Persistent diarrhea	2.20 CI95%: 0.53—9.06	-
Bacteremia	6.83 CI95%: 2.21—21.08	3.41 CI95%: 1.08—10.76
ESBL	1.09 CI95%: 0.13—8.70	-
ICU requirement	12.92 CI95%: 2.95—56.47	8.13 CI95%: 1.82—37.76

*ESBL* Extended spectrum β-lactamases, *ICU* intensive care unit

*PR* prevalence ratio, *aPR* adjusted prevalence ratio

## Discussion

An absence of information about the patterns of Salmonellosis in Latin America has been identified and an imperative need has arisen to generate population and clinical studies to evaluate disease dynamics, mortality and risk factors for severe disease [11, 19]. This study contributes to the generation of knowledge about clinical patterns, resistance, mortality and related factors in the region, in addition to providing a basis that can be used in future monitoring of the disease.

Although it was found an apparent low prevalence of salmonellosis with respect to the total number of health consultations in our hospital, 4% of the patients attended with this condition died. In addition, in patients with other associated conditions, such as bacteremia, infections by ESBL species, or neoplasms, mortality rises significantly to a proportion close to 10%. This situation is compounded by an increase in the rates of resistance to the first-line antibiotics commonly used for the treatment of the disease. According to these findings, salmonellosis remains a public health problem, especially for populations with vulnerable health conditions. Strengthening prevention policies, improving access to diagnostic tools in low-income countries, and promoting the responsible use of antibiotics in the treatment of infectious diseases should be a public health priority.

It was identified a similar proportion of Salmonella cases in children and adults, and in women and men. Previous studies have already reported that there are no significant differences in the proportion of cases by sex [20]; however, it is usually described that *Salmonella spp.* affects more children than adults and usually occurs in immunocompromised patients [21]. One of the conditions that may explain our high proportion of adults with Salmonellosis is the fact that our clinic receives a significant number of patients with malignant diseases, transplant recipients and/or immunosuppressed patients.

Consistent with what has been identified in previous studies [22], the most frequent symptoms of salmonellosis were diarrhea, fever, emesis and dehydration. Remarkably, one in five children aged 15 years or younger had respiratory symptoms, while abdominal pain occurred more frequently in patients older than 15 years. When identifying this type of symptomatology, especially in the context of foodborne disease outbreaks, it is important to suspect *Salmonella spp.* infections and to ask about risk factors such as not using a cutting board specifically for raw meat, consuming raw/undercooked meat or eggs, or the recent use of antibiotics [23].

If salmonellosis is suspected, the corresponding study and empirical management of the cases should be initiated according to the severity and diagnostic suspicion. Antimicrobial agents are not recommended for the treatment of non-severe NTS diarrhea in healthy adults or children; however, their use is indicated for persons with evidence of sepsis, extraintestinal infection, populations with bacteremia or risk of bacteremia, and patients with disseminated disease. Antibiotics such as ampicillin, amoxicillin, azithromycin, fluoroquinolones, 3rd and 4th generation cephalosporins or carbapenems can be used according to the conditions of the patients [24]. In our study, more than 70% of the patients received empirical antibiotic therapy. This can be explained by the fact that, being a highly complex institution, it is more likely to find patients with more severe conditions and greater underlying comorbidities, which is also supported by the fact that nearly 80% of the patients required in-hospital management.

Most of *Salmonella spp.* infections in which the species was identified in our population corresponded to NTS, with an approximate ratio of 9:1 respect to *Salmonella typhi* isolates. This is consistent with a high NTS prevalence described in food in Latin American studies, which ranged from 0.005% to 93.3%. Contrarily, previous reports of studies conducted in the old world have described a case distribution of 27.07% of NTS and 72.93% of *Salmonella typhi* [25]. The finding of a higher prevalence of NTS in our population is of special interest considering that the proportion of antimicrobial resistant isolates was higher in NTS than in *Salmonella typhi*.

Mortality in the group older than 15 years was found to be slightly higher than in the group aged 15 years or younger, although these differences did not reach statistical significance (5.4% vs. 2.8%; p 0.13). In a recently published systematic review with meta-analysis, no studies were found evaluating mortality by NTS in the Americas region in areas other than the United States that recorded higher mortality in people older than 15 years than that estimated in younger people (21% [16.6–25.7] vs. 12% [9–15.4%]) [11].

We hypothesize that the reason why our mortality was considerably lower than that estimated in that study may be because, being a highly complex institution, there may have been greater clinical suspicion, facilitating timely diagnosis. In addition, with greater opportunity for access to early antibiotic therapy directed against the infecting agent, compared to centers of lower complexity. Despite this, we found that mortality occurred mainly in vulnerable groups such as those with hematological malignancy, bacteremia and ICU requirement, so it is necessary to strengthen surveillance and therapeutic measures in these vulnerable groups. A recent study performed in oncologic patients with NTS infections found that salmonellosis produced more severe disease [26]. Although we did not find statistically significant associations in the multivariate model of hematological malignancy with mortality, we did find a higher proportion of deaths in that group. This may be due to lack of power due to poor representation of these subgroups in our cohort. Future studies with larger sample sizes are required to obtain better estimates.

One of the most important problems evidenced by our research is the increase in antibiotic resistance in recent years, which represents a threat to public health in our communities. According to our study, the most prevalent resistance phenotypes in *Salmonella spp.* are against ciprofloxacin, ceftriaxone, ampicillin/sulbactam and ampicillin, with a sustained increase in the resistance rates to these antibiotics in addition to cefepime. Some factors that could explain this increase are the indiscriminate use of self-formulated antibiotics, incomplete treatments by the patients, the use of broad-spectrum antibiotics in livestock and poultry, the use of chemicals such as triclosan, among others [27, 28].

Our findings are consistent with reports from the Centers for Disease Control and Prevention (CDC) indicating an increasing prevalence of antibiotic-resistant NTS infections, with resistance rates to any antibiotic reaching close to 20% in 2017 [29]. However, we observed a strikingly low proportion of ciprofloxacin-resistant *Salmonella typhi* samples in our population (less than 1%), despite that up to 74% of *S. typhi* strains were non-susceptible to this antibiotic in the CDC report. Instead, the highest resistance profiles were against TMP/SMX and ceftriaxone, with a high proportion of ESBL in our population. Notably, resistance to ceftriaxone was estimated to reach 60% in NTS. A previous study evaluating the geographic distribution and trends of typhoid fever disease in Colombia, based on data from the National Institute of Health, reported 836 isolates over a 3-year period, with a mortality rate of 0.74% and 84.3% of *S. typhi* sensitive to all antimicrobials. In agreement with our findings, in the INS study, resistance to ciprofloxacin was only 2% of the isolates [30].

Some of the limitations of this study are its retrospective nature, the use of medical records as a secondary source of information that in some cases may not contain all the information regarding the conditions of the patients at the time of infection, the lack of long-term follow-up of infected patients, the low availability of isolates serotyped for salmonella, and the selection and information biases inherent to this type of observational research. In addition, since the Salmonella cultures were performed according to medical criteria, it is possible that some cases of salmonellosis that consulted at Fundación Valle de Lili were not identified by our study. Also, it is necessary to consider the possibility of a beta error, as the “n” in some analysis performed was low. Among the strengths are the number of patients enrolled, the efforts to ensure better data quality through double validation and structured data entry in electronic case report formats, the use of standardized definitions and trained personnel for the identification of information in the clinical history, and the fact of discriminating the analysis by clinical groups of interest and by age, which can be a source of confusion in less robust analyses.

## Conclusions

The proportion of cases was similar in both genders, with greater involvement in pediatric patients. The most common clinical presentation was gastroenteric syndrome and bacteremia. The presence of bacteremia and ICU requirement were associated with a higher probability of death in adults. A sustained increase in the resistance rates to ciprofloxacin, cefepime, ampicillin and ampicillin/sulbactam was recorded in the last 10 years. Salmonellosis remains a public health problem. It is necessary to strengthen prevention policies, improve access to diagnostic tools in low-income countries and promote the responsible use of antibiotics.

### Supplementary Information

**Additional file 1: Table S1. **Proportion of resistant isolates by species and antibiotic during the study period.

## Data Availability

The data and analytic code will be shared at any time, upon request to the corresponding author, with investigators whose proposed use of the data has been approved by an independent review committee.

## Abbreviations

aPR: Adjusted prevalence ratio
CNA: Columbia Nalidixic Acid
CNPt: CARBA NP test
CLSI: The Clinical & Laboratory Standards Institute
ESBL: Extended spectrum β-lactamases
FVL: Fundación Valle de Lili
HEA: Hektoen enteric agar
HIV: Human Immunodeficiency virus
ICU: Intensive Care Unit
IQR: Interquartil range
MAC: MacConkey agar
Me: Median
NTS: Non-typhoideal Salmonellae
PR: Prevalence ratio
TMP/SMX: Trimethoprim / sulfamethoxazole
