# Supplementary material for: Clinical and microbiological characterization of Salmonella spp. isolates from patients treated in a university hospital in South America between 2012–2021: a cohort study
Source: BMC Infect Dis. 2023 Sep 25;23:625. doi: 10.1186/s12879-023-08589-y (PMC10519077; doi:10.1186/s12879-023-08589-y)
Supplement: Supplementary file 1 — Additional file 1: Table S1. Proportion of resistant isolates by species and antibiotic during the study period. [file 12879_2023_8589_MOESM1_ESM.docx]

**SUPPLEMENTARY MATERIAL**

**Table S1. Proportion of resistant isolates by species and antibiotic during the study period.**

| **Antibiotic tested in antibiogram** | ***Salmonella spp.***   **resistant isolates** | ***Salmonella no typhi***   **resistant isolates** | ***Salmonella typhi* resistant isolates** |
| --- | --- | --- | --- |
|  | **N=405** | **N=99** | **N=14** |
| Nalidixic acid, n=21 | 16.67% | <1% | <1% |
| TMP/SULFA, n= 431 | 18.29% | 11.7% | 22.22% |
| Ciprofloxacin, n= 431 | 14.13% | 59.09% | <1% |
| Ceftriaxone, n= 261 | 19.00% | 14.29% | 60% |
| Ampicillin, n= 457 | 21.19% | 30.53% | <1% |
| Ampicillin/sulbactam, n= 313 | 12.58% | 16.67% | <1% |
| Ceftazidime/avibactam, n= 125 | 7.45% | 3.57% | <1% |
| Ertapenem, n= 218 | 1.08% | <1% | <1% |
| Meropenem, n= 229 | 0.52% | <1% | <1% |
| Piperacillin/tazobactam, n= 104 | 4.25% | <1% | <1% |
| Cefepime, n=219 | 6.42% | 12.5% | <1% |
| ESBL, n= 381 | 13.13% | 9.26% | 42.86% |
| CNPt, n= 307 | 0.44 % | <1% | 11.11% |
| CARBA-5, n= 305 | <1% | <1% | 11.11% |
| CNPt: CARBA-NP test. |  |  |  |
